# Project orange elephant is a conflict specific holistic approach to mitigating human-elephant conflict in Sri Lanka

**DOI:** 10.1038/s42003-020-0760-4

**Published:** 2020-01-23

**Authors:** Chathuranga Dharmarathne, Chandima Fernando, Chinthaka Weerasinghe, Ravi Corea

**Affiliations:** Sri Lanka Wildlife Conservation Society, 6/5 Kanatte Road, Udahamulla, Nugegoda 10250 Sri Lanka

**Keywords:** Conservation biology, Agriculture, Policy

## Abstract

Human-wildlife conflicts are an increasing problem as human land use encroaches on wildlife habitats. Augmenting farmers’ crops with orange trees through Project Orange Elephant has proven to be a simple and effective method for mitigating human-elephant conflicts in Sri Lanka. Similar endeavours could be applied elsewhere in the world.

## Abstract (web summary)

Chathuranga Dharmarathne et al. discuss an innovative conservation project to reduce human-elephant conflict in Sri Lanka. This project uses citrus trees to repel elephants from farms, thereby reducing crop loss among farmers and providing additional income.

## The problem

The association between man and elephant in Sri Lanka is ancient and dates back nearly 5000 years. The Asian elephant (*Elephas maximus*), being the largest terrestrial herbivore on the island, naturally requires large and diverse habitats to survive. With human expansion comes land modification, unfortunately to the detriment of elephants. The need of land for human use is an ongoing encroachment of the existing elephant habitat which is being diminished continuously and drastically. As a result, Human–Elephant Conflict (HEC) is escalating every year in frequency and intensity. Annually ~250 elephants and ~80 people are killed due to HEC.

Agriculture is the primary rural industry and rice is the staple food in Sri Lanka. Most rice cultivators are small-scale farmers and they are the people who suffer frequently from HEC. These farmers are hampered by poor economies and financial services, limited technology, fragmented landholding, and pre and post-harvest losses, and HEC.

Conflict with elephants continue to increase due to inefficient landscape-level planning and land-use practices that are incompatible to coexisting with elephants. Currently, there are very few efforts to develop solutions to resolve the livelihood and environmental concerns resulting due to the negative interactions of agriculture and elephants. Since HEC being a result of agriculture-based land used practices incompatible with elephants, a large part of the solution to mitigate HEC must be based on the introduction of innovative land-use practices.

## The solution

**Fig. 1:**
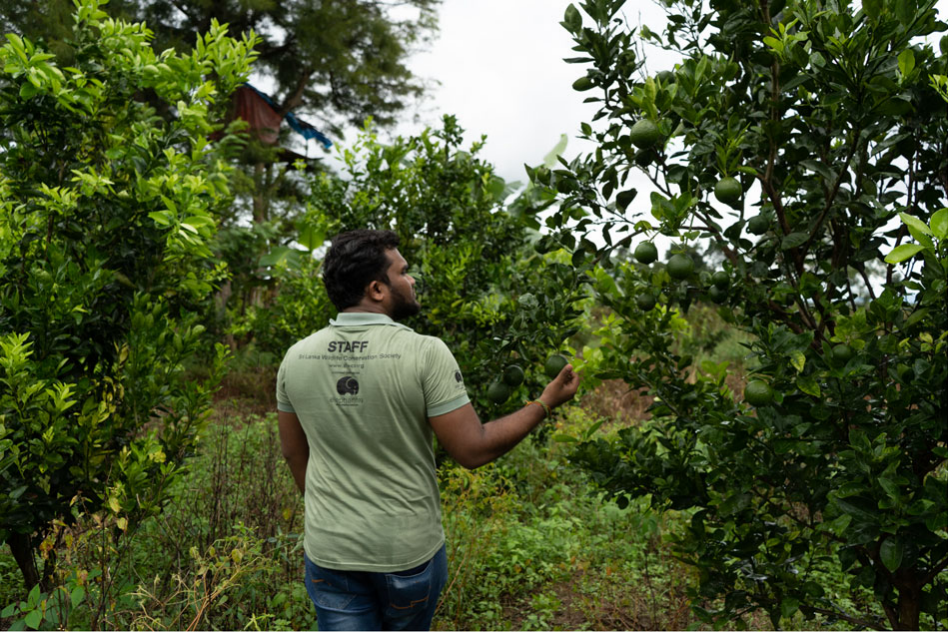
Staff and volunteers collaborate with communities and empower citizens of Sri Lanka to support sustainable, long-term conservation projects.

Project Orange Elephant (POE) is an innovative initiative conceptualized by the Sri Lanka Wildlife Conservation Society (SLWCS) (Fig. 1). The initiative draws on elephants’ natural aversion to citrus to protect the homes of farmers from elephant attacks while at the same time providing farmers with a sustainable supplementary income. The project is unique to Sri Lanka and came about as the result of fieldwork conducted by the SLWCS in the Wasgamuwa area of the Central Province of Sri Lanka.

Elephants do not preferentially eat oranges. To test this observation a series of feeding trials were conducted with six Asian elephants at the Sri Lanka National Zoological Gardens in 2006. The results were very encouraging, the elephants showed an obvious distaste for oranges and several other citrus varieties.

**Fig. 2:**
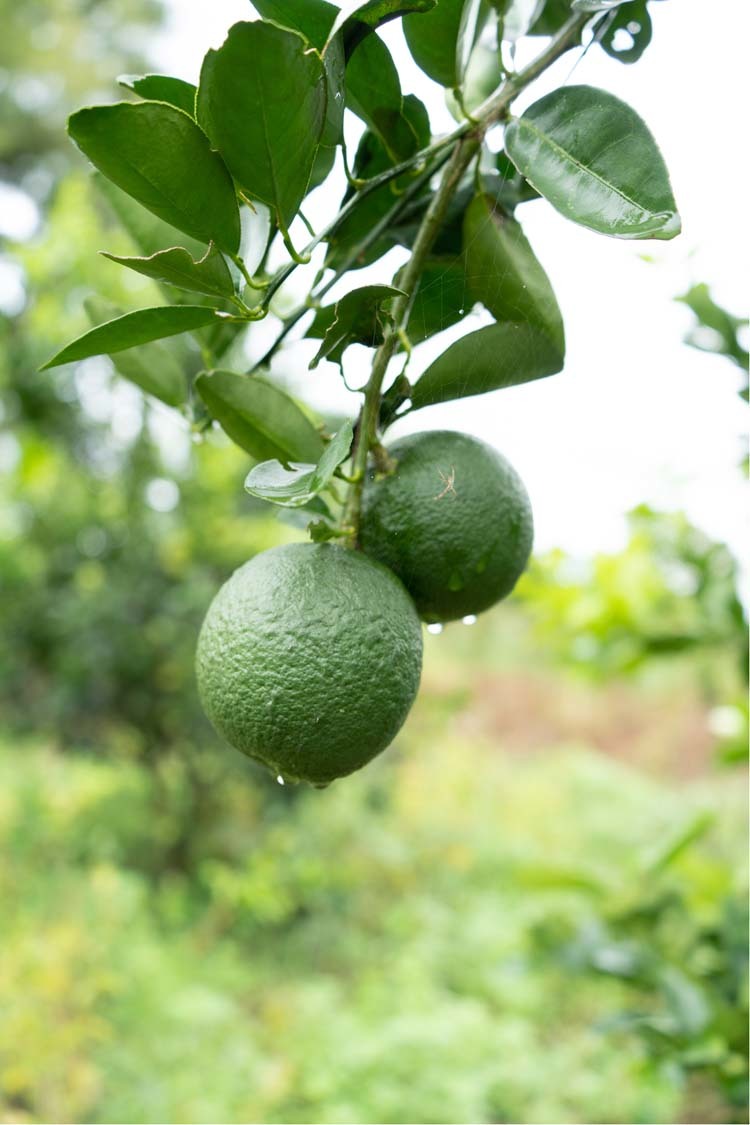
Orange trees mask the smell of crops and provide a natural deterrent that helps prevent elephants from raiding farms, while also providing a supplemental income for farmers.

POE is a crop diversification project to establish an economic and trophic buffer for farmers living in the rural countryside for which close encounters with elephants during their day-to-day activities are commonplace. Crop raiding by elephants and the harsh retaliatory measures subsequently taken by people whose livelihoods depend on their farms feeds a vicious cycle of violence and death. POE is helping to reduce this violence. The project uses a variety of grafted local orange (*Citrus sinensis*) known as Bibile Sweet that had been developed in Sri Lanka to suit the local climatic conditions (Fig. 2). This variety grows well in marginalized land and does not need frequent irrigation, bears fruit within 1.5–3 years, and each tree can provide ~300–600 fruits per season. Each tree has a fruiting cycle of two seasons per year. Bibile Sweet is a high-quality fruit known for its fresh sweetness, value-added products, and has good market demand. By planting these trees alongside their crops, particularly in the border areas adjacent to elephant habitats, we hope to decrease crop raiding by elephants while also supplementing farmers’ income

The objective of POE is to develop solutions at the micro-level to have an impact at the macro level to reduce HEC through a better understanding of elephant biology, ecology, behaviour, human-needs, and aspirations. By mobilizing local communities to grow oranges as an alternative cash crop the project provides assistance to elevate them socio-economically by providing them with a sustainable primary income from cultivating oranges. The SLWCS hopes to encourage more and more farmers to join the project to provide a sustainable solution to reduce HEC and create an environment of coexistence by scaling up POE. This effort will not only help to mitigate HEC but also alleviate rural poverty helping to garner the support of local communities for the long-term conservation of the endangered Sri Lankan elephant.

**Fig. 3:**
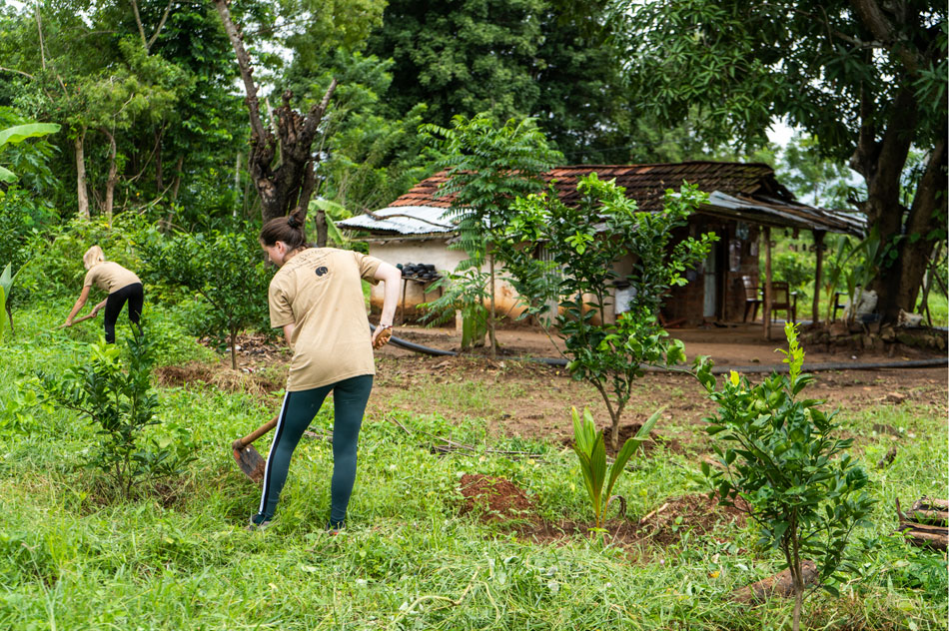
SLWCS runs on volunteers, who work hand-in-hand with local staff to carry out essential conservation work.

The key factors of the project is its simplicity. The project does not involve advance technological transfers or teaching farmers brand new skills or building their capacity to do something other than what they are traditionally trained to do. The project is harnessing farmers’ existing skills and abilities to do something new and economically, socially and ecologically beneficial to them and the environment. For the most part, POE can be managed totally with community resources. SLWCS encourage farmers to cultivate crops such as oranges that are not attractive to elephants, rather than rice cultivation. The Society believes POE can change the future of elephants and farmers. If we can get these rice farmers to cultivate oranges at least in the border areas adjacent to elephant habitats then these farmers will have a primary income that is not susceptible to elephant depredations. This will contribute to creating coexistence in a landscape where people and elephants share space. For more than a decade the SLWCS has been working with local communities to create alternative forms of agriculture and to create livelihoods that cannot be destroyed by elephants (Fig. 3). By cultivating oranges, rural Sri Lankan farmers in Wasgamuwa are creating an effective elephant trophic deterrent and a sustainable income for themselves while at the same time ensuring the safety of their families and the conservation of the endangered Sri Lankan elephant.

In 2015, Project Orange Elephant received a Most Innovative Development Project Award from the Global Development Network (GDN) based in Washington, DC, U.S.A. For more information on the project, you can visit our website (https://www.slwcs.org/project-orange-elephant).

Saving Elephants by Helping People is the underlying and overlying philosophy as well the goal and objective of the Sri Lanka Wildlife Conservation Society’s efforts to develop holistic and sustainable measures to mitigate human–elephant conflict in Sri Lanka.

